# Variation in the Occurrence of *fimA* Genotypes of *Porphyromonas gingivalis* in Periodontal Health and Disease

**DOI:** 10.3390/ijerph17061826

**Published:** 2020-03-11

**Authors:** Manohar Kugaji, Uday Muddapur, Kishore Bhat, Vinayak Joshi, Manjunath Manubolu, Kavitha Pathakoti, Malleswara Rao Peram, Vijay Kumbar

**Affiliations:** 1 Central Research Laboratory, Maratha Mandal’s NGH Institute of Dental Sciences & Research Centre, Bauxite Road, Belagavi, Karnataka 590010, India; manoharkugaji@gmail.com (M.K.); pvsnmallesh@gmail.com (M.R.P.); vijaykumbarbtg@gmail.com (V.K.); 2 B.V. Bhoomaraddi College of Engineering and Technology, KLE Technological University, Vidya Nagar, Hubballi, Karnataka 580031, India; muddapur@kletech.ac.in; 3 Division of Periodontology, College of Dentistry, The Ohio State University, Columbus, OH 4312, USA; drvinayakjoshi@gmail.com; 4 Aquatic Ecology Laboratory, Department of Evolution, Ecology and Organismal Biology, The Ohio State University, Columbus, OH 4312, USA; manubolu.1@osu.edu; 5 Interdisciplinary Center for Nanotoxicity, Department of Chemistry, Physics and Atmospheric Sciences, Jackson State University, Jackson, MS 39217, USA; kavitha.pathakoti@jsums.edu; 6 Department of Biology, Jackson State University, Jackson, MS 39217, USA

**Keywords:** fimbriae, polymerase chain reaction (PCR), periodontitis, *Porphyromonas gingivalis*, virulence

## Abstract

*Porphyromonas gingivalis* is regarded as a “keystone pathogen” in periodontitis. The fimbria assists in the initial attachment, biofilm organization, and bacterial adhesion leading to the invasion and colonization of host epithelial cells. The present study aimed to investigate the occurrence of *fimA* genotypes in patients with chronic periodontitis and healthy individuals in the Indian population, and to study their association with the number of *P. gingivalis* cells obtained in subgingival plaque samples of these subjects. The study comprised 95 samples from the chronic periodontitis (CP) group and 35 samples from the healthy (H) group, which were detected positive for *P. gingivalis* in our previous study. Fimbrial genotyping was done by PCR and PCR-restriction fragment length polymorphism (RFLP). The *fimA* type II was more prevalent in the CP group (55.89%), followed by type IV (30.52%), whereas in the H group, type I was the most prevalent fimbria (51.42%). The quantity of *P. gingivalis* cells increased with the presence of *fimA* types II and III. Our results suggest a strong relationship between *fimA* types II and IV and periodontitis, and between type I and the healthy condition. The colonization of organisms was increased with the occurrence of type II in deep periodontal sites, which could play an important role in the progression of the disease.

## 1. Introduction

*Porphyromonas gingivalis* is a Gram-negative, black-pigmented, anerobic bacterium responsible for causing chronic periodontitis by establishing itself in the deep periodontal pockets of the oral cavity [1]. *P. gingivalis* is regarded as a “keystone pathogen” in periodontitis and plays an essential role in its pathogenesis [2]. Apart from playing a significant role in periodontitis, *P. gingivalis* is also known to be a risk factor for cardiovascular disease, type 2 diabetes mellitus, and Alzheimer’s disease through immune modification mechanisms [3,4,5]. It is reported that *P. gingivalis* is not only abundantly present in chronic periodontitis patients, but is also known to occupy the oral cavity of healthy individuals. The pathogenicity of *P. gingivalis* is ascribed to diverse virulence factors, such as fimbriae, lipopolysaccharides, membrane proteins, capsules, proteases, endotoxins, cysteine proteases, etc. [6]. Fimbriae are arranged on the cell surface of the pathogen and play a vital role in initial attachment and biofilm organization. The bacteria adhere to gingival tissue leading to an invasion and colonization of host epithelial cells [7]. These structures are also known to induce cellular activation and cytokine release, thereby producing an inflammatory response at the infected site [8]. Based on the nucleotide variation, fimbriae are classified into six genotypes (type I, Ib, II, III, IV and V), and are encoded by the *fimA* gene [9]. Studies have been carried out to examine the presence of these genotypes in different geographical locations and ethnic groups to investigate the relationship between the *fimA* genotype and disease severity [9,10,11,12,13,14,15]. In Japanese adults, types II and IV have been found to be more prevalent in periodontitis patients, whereas type I has been associated with healthy individuals [9]. In Caucasians, types II and I are more prevalent in periodontitis patients, whereas in Brazilian periodontitis patients, types II and Ib were found to be more prevalent [11,15]. These findings suggest that variability in the frequency of fimbriae could be related to geographical location, ethnic group, and periodontal health status.

Until now, there have been no reports on the complete genotyping of the fimbriae of *P. gingivalis* in Indian subjects. The present study was aimed to investigate the prevalence of *fimA* genotypes in patients with chronic periodontitis and healthy individuals, in relation to clinical parameters, in the Indian population. We also aimed to study the association of *fimA* genotype with the number of *P. gingivalis* cells obtained in the subgingival plaque samples of these subjects.

## 2. Materials and Methods 

The study comprised 95 samples from the chronic periodontitis (CP) group and 35 samples from the healthy (H) group, which were detected positive for *P. gingivalis* among 120 samples from each group in our previous study [16]. The subjects were separated into the CP and H groups as per the American Association of Periodontology guidelines [17]. Subjects with diabetes, HIV infection, pregnant women, and those on any medication or antibiotic therapy were excluded from the study. Subjects with at least 20 teeth with gingival inflammation, bleeding on probing, probing depth of ≥5 mm, and clinical attachment loss of ≥3 mm in at least four siteswere included in the CP group. Subjects with no signs of gingival inflammation, absence of bleeding on probing, probing depth of ≤3 mm, and no clinical attachment loss in all the sites were included in the H group. 

### 2.1. Sample Collection

Subgingival plaque samples were collected and pooled using a dental curette as described in our previous study [18]. Briefly, six sites having the deepest probing depths were selected. Sites were isolated with sterile cotton rolls and air dried, and the supragingival plaque was removed. The subgingival sample was collected and pooled in a vial containing Tris EDTA buffer using a dental curette. Clinical parameters such as gingival index (GI) [19], plaque index (PI) [20], probing depth (PD), and clinical attachments loss (CAL) were averaged for each individual in both the healthy and CP groups.

### 2.2. Polymerase Chain Reaction (PCR)

DNA was extracted from subgingival plaque samples by the “modified proteinase K” method as described previously [21]. A real time polymerase chain reaction (RT-PCR) was carried out for amplification of the 16S rRNA species-specific gene of *P. gingivalis* in our previous study [16]. Samples found to be positive for *P. gingivalis* were further processed for the detection of *fimA* genotypes I to V by polymerase chain reaction (PCR), as described previously [22,23]. The primers specific to each of the fimA types used in the study were as mentioned in Table 1. A reaction mixture with a total volume of 25 µL was prepared by using Ampliqon red 2X mastermix (Ampliqon, Odense, Denmark), which contains Tris-HCL pH 8.5, (NH_4_)_2_SO_4_, 3 mM MgCl_2_, 0.2% Tween 20, 0.4 mM of each dNTP, 0.2 units/µL Ampliqon Taq DNA polymerase, and inert red dye and stabilizer. The primers were used at 0.5 µM concentration, and 3 µL of the DNA template at approximately 100ng concentration was added to the reaction mixture. The thermal cycling conditions were performed in a veriti 96-well thermal cycler (Applied Biosystems, California, CA, USA). An initial denaturation was done at 95 °C for 5 min, followed by 35 cycles of 95 °C, 58 °C and 72 °C for 30 s each. The final extension was carried out at 72 °C for 5 min.

### 2.3. Restriction Fragment Length Polymorphism (RFLP)

The samples which were found positive for both type I and type II concurrently were further processed for restriction fragment length polymorphism. The primer pair Ib was used for PCR amplification, and the amplified products were digested with 10 u/µL of FastDigest RsaI enzyme. The reaction mixture was incubated at 37 °C for 5 min [10].

PCR amplified products and restriction digested products were subjected to 2% agarose gel electrophoresis using Tris-acetate EDTA buffer at 80 volts for 2 h. The gel was stained with 0.5 µg/mL of ethidium bromide and observed under a UV gel documentation system (Major Science, Saratoga, CA, USA). The specific fimA type was identified by comparing the band size of each sample with a 100 bp DNA ladder.

### 2.4. Statistical Analysis 

Statistical analysis was carried out by using GraphPad Prism 5.1 (GraphPad Software, Inc., San Diego California, CA, USA). The frequency of fimA genotypes and their statistical association with chronic periodontitis was evaluated by using Fisher’s exact test. Association of *fimA* genotypes with clinical parameters and the quantity of *P. gingivalis* was analyzed by the unpaired *t*-test and the Mann–Whitney U test, respectively. *p* < 0.05 was considered as statistically significant.

## 3. Results

**Figure 1:**
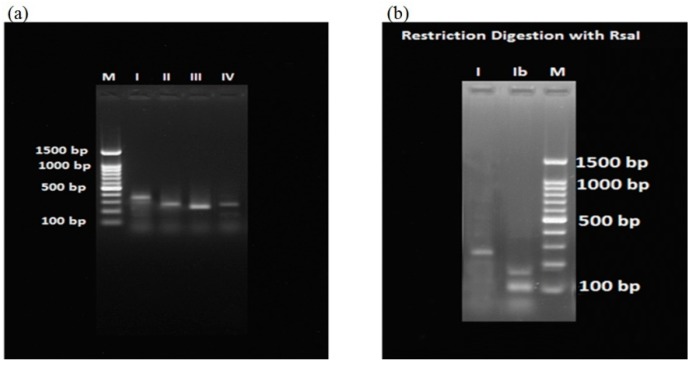
Agarose gel images showing the amplification of *fimA* genotypes: (**a**) amplification of *fimA* type I (392 bp), type II (257 bp), type III (247 bp), and type IV (251 bp); (**b**) restriction digestion with the RsaI enzyme yielded type I (271 bp) and type Ib (169 bp, 109 bp).

**Figure 2:**
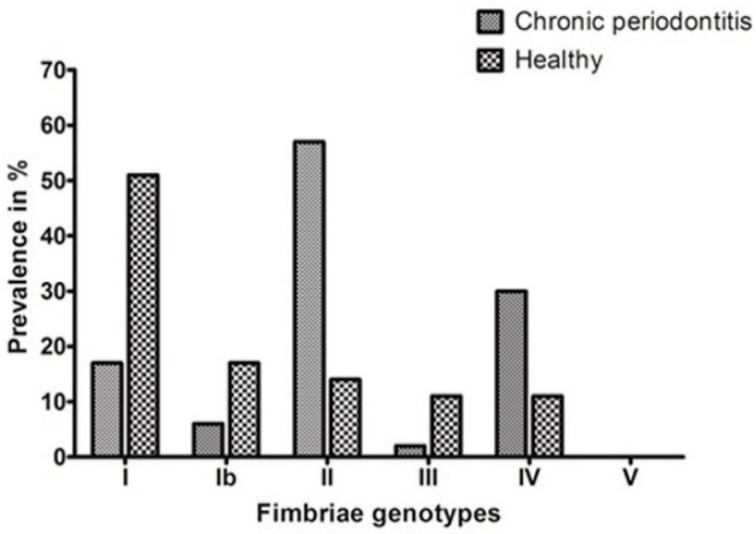
Bar chart showing the frequency distribution of *fimA* genotypes in subjects detected positive for *P. gingivalis* in the chronic periodontitis and healthy groups.

This is a continuation study, which we initiated on 120 samples each from the CP group and H group, for quantitative detection of *P. gingivalis* in subgingival plaque samples by real time PCR in Indian subjects [16]. In the present study, 95 samples positive for *P. gingivalis* in the CP group and 35 samples positive in the H group were subjected to fimbrial genotyping by the PCR method. The detection of specific amplified products of *fimA* genotypes is as shown in Figure 1. The frequency of distribution of these *fimA* genotypes in the CP and H groups is as shown in Table 2 and Figure 2. Among these genotypes, type II was the most prevalent type in the CP group, followed by type IV, with prevalence rates of 55.89% and 30.52%, respectively. The *fimA* types I, Ib, and III were at lesser frequencies of 17.89%, 6.31% and 2.1%, respectively. Type V was not detected in the CP group. There was also a co-existence of two *fimA* types concurrently, with type II and type IV being present in most of the cases. Type II and type IV were significantly associated with the CP group (OR 8.250 and 3.405, respectively) with a *p*-value < 0.0001 and 0.0393, respectively. 

In healthy individuals, type I was the most prevalent fimbria, found in 51.42% of cases. Type Ib and type II were found in 17.14% and 14.28% of *P. gingivalis* positive cases. Type III and type IV were found at a rate of 11.4%, and type V was not found in any of the samples. Co-existence of types I and II was found in 2.8% of cases, and types I and IV were found at the same rates. Type I was found to be significantly associated with the H group (*p*-value = 0.0003).

The association of these *fimA* genotypes with clinical parameters in CP is as shown in Table 3. When the comparison was made between the groups with the presence of different genotypes, the mean probing depth was found to be higher (5.76 ± 0.62) for *fimA* type II when compared to other types, except for type III (6.55 ± 1.06), which was detected in just two samples. The clinical attachment level was also found to be marginally increased (5.40 ± 1.04) with the presence of *fimA* type II, whereas the probing index (2.52 ± 0.19) and gingival index (2.57 ± 0.21) were found to be highest with the presence of *fimA* type IV.When the comparison was made within the groups for the presence and absence of different genotypes, probing depth was found to be significantly associated with the presence of type III *fimA* (*p*-value = 0.03), whereas the clinical attachment level was significantly associated with the presence of type II *fimA* (*p*-value = 0.04).

The association of *fimA* genotypes with the quantities of *P. gingivalis* is as shown in Table 4. The Mann–Whitney U test was performed to report the difference in the quantities of *P. gingivalis* obtained in the presence or absence of a specific genotype. The quantity of *P. gingivalis* was significantly higher in the *fimA* type II (2.09 × 10^8^) positive samples than in the negative samples (9.52 × 10^7^). The difference was statistically significant with a *p*-value < 0.001. We also found an increased mean cell count of *P. gingivalis* in the positive samples of fimA type III (2.77 × 10^9^) compared to the type III negative samples (1.03 × 10^8^). The difference was statistically significant with the *p*-value = 0.0385. 

## 4. Discussion

In this study, we have analyzed the association of the presence of fimbrial genotypes with chronic periodontitis and periodontal health status in Indian subjects. In healthy individuals, type I was found to be more prevalent, followed by types Ib and II.The prevalence of *fimA* types II and IV was found to be higher in the CP group, followed by type I. The prevalence rate of fimA types II and IV in the CP group from our study was in accordance with another previous report carried out on Japanese and Chinese patients [9,13]. This was also in agreement with other studies carried out on Caucasian and Swedish periodontitis patients [15,24]. We also detected *fimA* type I in greater frequency in periodontitis cases, which is well in accordance with Beikler et al., rather than with other previous reports. Interestingly, Missailidis CG et al. carried out a study in Brazilian patients and found a higher prevalence of type Ib than type IV [11]. The difference in the detection frequencies could be due to the different geographical location, ethnic source, and technical variability. 

A study conducted by Zhao et al. showed that genotypes II and IV were more frequently detected in 4–6 mm and >7 mm probing depth groups among patients with periodontitis [13]. Our report also showed that the mean probing depth and clinical attachment loss were marginally higher with the presence of type II fimbriae, whereas the probing index and gingival index were found to be higher with the presence of type IV fimbriae. This suggests the possible role of type II and type IV fimbriae in the initiation and progression of periodontitis in Indian subjects. It could be interesting to see the association of the fimbrial genotype with the different age groups, which we could not do in our study due to the small sample size. Previous reports have suggested that a certain type of relationship does exists between the *fimA* genotype and age [9,11,15,25].

The prevalence of the *fimA* genotype was also correlated with the quantity of *P. gingivalis* obtained by the real time PCR technique. When the comparison was made between genotypes, the cell count of *P. gingivalis* was greater with the presence of *fimA* type III, but this type was detected in just two samples. Meanwhile, within the groups, the level of *P. gingivalis* was found to be significantly higher in the presence of *fimA* types II and III (*p*-value < 0.001 and 0.0385, respectively). Although a greater number of samples in *fimA* type III cases would have provided a clearer idea on its role in the pathogenesis, nevertheless, our study underlines the significant role of *fimA* type II in the colonization of *P. gingivalis* in the oral cavity. Miura et al. found that a *P. gingivalis* isolate with type I *fimA* had a higher Arg- and Lysine-gingipain activity, suggesting dual-action attachment to gingival tissue by a fimbrial mechanismand destruction by proteinase enzyme activity [12]. This relationship can be further investigated by studying other fimbrial types with other possible virulent mechanisms. 

There are two limitations of this study. Small numbers of samples were detected for *fimA* genotypes (N = 2 for *fimA* type III, and N = 6 for *fimA* type Ib). A higher number of samples in the original study itself probably would have provided more numbers of samples for *fimA* genotypic analysis, in order to draw a fair conclusion for all the genotypes simultaneously. Another limitation was that pooled samples were collected, and therefore the co-existence of different *fimA* genotypes in the same ecological niche could not be analyzed. In order to relate colonization to a particular ecological niche by a certain genotype, the individual site needs to be assessed.

With the dominance of a specific fimbrial type in the healthy and periodontitis groups, it is evident that variability in the presence of the fimbrial genotype could possibly provide variable pathogenic ability to different *P. gingivalis* clonal types across all geographic locations. Also, along with the fimbriae, there are other virulence factors, such as outer membrane proteins, capsules, proteases, endotoxin lipopolysaccharides, cytotoxic metabolites, etc. which may act concomitantly to provide differential pathogenic abilities to the *P. gingivalis* organism. Recently, studies on periodontitis have been focused on the inhibition of the virulence potential of *P. gingivalis* by different mechanisms to provide health benefits in these subjects [26,27].The variable pathogenic ability due to fimbrial occurrences could also lead us to organize primary and secondary prevention systems and suggests the use of fimbrial proteins, such as fimbrillins in immunoprophylaxis or immunotherapeutic strategies, to eliminate this pathogen from the subgingival microbiota.

## 5. Conclusions

Our results clearly suggest that there is strong relationship between *fimA* types II and IV and periodontitis, and that type I is associated with the healthy condition. The study also demonstrated that with the presence of *fimA* type II, there was increased colonization of *P. gingivalis* organisms in deep periodontal sites, which could play an important role in the progression of the disease. 

## Figures and Tables

**Table 1:** PCR primers used in the study.

*fimA* Type	Primer Sequence 5′-3′	Amplification Length (bp)	Reference
I	CTG TGT GTT TAT GGC AAA CTT C AAC CCC GCT CCC TGT ATT CCG A	392	[22]
Ib	CAG CAG AGC CAA AAA CAA TCG TGT CAG ATA ATT AGC GTC TGC	271	[10]
II	ACA ACT ATA CTT ATG ACA ATG G AAC CCC GCT CCC TGT ATT CCG A	257	[22]
III	ATT ACA CCT ACA CAG GTG AGG C AAC CCC GCT CCC TGT ATT CCG A	247	[22]
IV	CTA TTC AGG TGC TAT TAC CCA A AAC CCC GCT CCC TGT ATT CCG A	251	[22]
V	AAC AAC AGT CTC CTT GAC AGT G TAT TGG GGG TCG AAC GTT ACT GTC	462	[23]

**Table 2:** Frequency distribution of *fimA* genotypes in subjects detected positive for *P. gingivalis* in the chronic periodontitis and healthy groups.

Factor	CP Group		H Group		Odds Ratio	95% Confidence Interval	*p*-Value
	N	%	N	%			
I	17	17.89	18	51.42	0.20	0.0884 to 0.4794	0.0003 *
Ib	6	6.31	6	17.14	0.3258	0.09746 to 1.089	0.08
II	55	57.89	5	14.28	8.250	2.943 to 23.13	<0.0001 *
III	2	2.1	4	11.4	0.1667	0.02908 to 0.9551	0.0446 *
IV	29	30.52	4	11.4	3.405	1.101 to 10.54	0.0393 *
V	0	0	0	0	-	-	-
I, II	5	5.26	1	2.8	1.889	0.2128 to 16.77	1.0000
Ib, II	3	3.1	0	0	-	-	-
II, IV	4	4.2	0	0	-	-	-
I, IV	2	2.1	1	2.8	0.7234	0.06350 to 8.241	1.0000

CP: chronic periodontitis, H: healthy individuals. * *p* < 0.05.

**Table 3:** Association of *fimA* genotypes with clinical parameters in patients with chronic periodontitis.

*fimA* Genotype	Present/Absent	PD		CAL		PI		GI	
		Mean ± SD	*p*-Value	Mean ± SD	*p*-Value	Mean ± SD	*p*-Value	Mean ± SD	*p*-Value
I	Present	5.54 ± 1.14	0.18	5.22 ± 1.06	0.96	2.45 ± 0.21	0.2	2.46 ± 0.26	0.21
	Absent	5.75 ± 0.66		5.21 ± 1.08		2.52 ± 0.19		2.54 ± 0.24	
Ib	Present	5.56 ± 0.27	0.51	4.98 ± 0.54	0.6	2.46 ± 0.22	0.53	2.51 ± 0.22	0.85
	Absent	5.72 ± 0.58		5.22 ± 1.09		2.51 ± 0.19		2.53 ± 0.25	
II	Present	5.76 ± 0.62	0.32	5.40 ± 1.04	0.04 *	2.50 ± 0.19	0.73	2.50 ± 0.27	0.18
	Absent	5.64 ± 0.49		5.02 ± 1.03		2.52 ± 0.19		2.56 ± 0.21	
III	Present	6.55 ± 1.06	0.03 *	6.1 ± 0.42	0.24	2.45 ± 0.07	0.64	2.55 ± 0.35	0.92
	Absent	5.69 ± 0.55		5.19 ± 1.07		2.51 ± 0.20		2.53 ± 0.24	
IV	Present	5.56 ± 0.39	0.08	5.15 ± 1.07	0.75	2.52 ± 0.19	0.86	2.57 ± 0.21	0.36
	Absent	5.78 ± 0.62		5.23 ± 1.07		2.51 ± 0.20		2.51 ± 0.26	

PD: probing depth, CAL: clinical attachment level, PI: plaque index, GI: gingival index, SD: standard deviation, unpaired *t*-test.* *p* < 0.05.

**Table 4:** Association of *fimA* genotypes with the quantity of *P. gingivalis* in patients with chronic periodontitis.

*fimA* Types	Positive/ Negative	N	Mean	± SEM	Median	Interquartile Range (IQR)	*p*-Value
I	Positive	17	2.35 × 10^7^	1.30 × 10^7^	4.53 × 10^6^	1.13 × 10^7^	0.1185
	Negative	103	1.68 × 10^8^	7.45 × 10^7^	1.58 × 10^6^	9.74 × 10^6^	
Ib	Positive	6	6.27 × 10^6^	1.61 × 10^6^	6.56 × 10^6^	8.12 × 10^6^	0.1894
	Negative	114	1.55 × 10^8^	6.74 × 10^7^	1.35 × 10^6^	1.02 × 10^7^	
II	Positive	55	2.09 × 10^8^	9.76 × 10^7^	4.61 × 10^6^	1.26 × 10^7^	<0.0001 *
	Negative	65	9.52 × 10^7^	8.49 × 10^7^	9.17 × 10^4^	5.79 × 10^6^	
III	Positive	2	2.77 × 10^9^	2.75 × 10^9^	2.77 × 10^9^	5.50 × 10^9^	0.0385 *
	Negative	118	1.03 × 10^8^	4.62 × 10^7^	1.59 × 10^6^	9.84 × 10^6^	
IV	Positive	29	1.49 × 10^8^	1.39 × 10^8^	2.08 × 10^6^	6.98 × 10^6^	0.273
	Negative	91	1.47 × 10^8^	7.25 × 10^7^	1.52 × 10^6^	1.04 × 10^7^	

N: number, SEM: standard error of the mean.* *p* < 0.05.
